# KHDRBS3 regulates the permeability of blood–tumor barrier via cDENND4C/miR-577 axis

**DOI:** 10.1038/s41419-019-1771-2

**Published:** 2019-07-11

**Authors:** Peiqi Wu, Yang Gao, Shuyuan Shen, Yixue Xue, Xiaobai Liu, Xuelei Ruan, Lianqi Shao, Yunhui Liu, Ping Wang

**Affiliations:** 1 Department of Neurobiology, School of Life Sciences, China Medical University, 110122 Shenyang, China; 2 Key Laboratory of Cell Biology, Ministry of Public Health of China, China Medical University, 110122 Shenyang, China; 3 Key Laboratory of Medical Cell Biology, Ministry of Education of China, China Medical University, 110122 Shenyang, China; 4 Department of Neurosurgery, Shengjing Hospital of China Medical University, 110004 Shenyang, China; 5 Liaoning Clinical Medical Research Center in Nervous System Disease, 110004 Shenyang, China; 6 Key Laboratory of Neuro-oncology in Liaoning Province, 110004 Shenyang, China

**Keywords:** miRNAs, CNS cancer

## Abstract

The existence of blood–tumor barrier (BTB) severely restricts the efficient delivery of antitumor drugs to cranial glioma tissues. Various strategies have been explored to increase BTB permeability. RNA-binding proteins and circular RNAs have recently emerged as potential regulators of endothelial cells functions. In this study, RNA-binding protein KH RNA-binding domain containing, signal transduction associated 3 (KHDRBS3) and circular RNA DENND4C (cDENND4C) were enriched in GECs. KHDRBS3 bound to cDENND4C and increased its stability. The knockdown of cDENND4C increased the permeability of BTB via downregulating the expressions of tight junction-related proteins. The miR-577 was lower expressed in GECs. The overexpressed miR-577 increased the permeability of BTB by reducing the tight junction-related protein expressions, and vice versa. Furthermore, cDENND4C acted as a molecular sponge of miR-577, which bound to miR-577 and inhibited its negative regulation of target genes ZO-1, occludin and claudin-1 to regulate BTB permeability. Single or combined treatment of KHDRBS3, cDENND4C, and miR-577 effectively promoted antitumor drug doxorubicin (DOX) across BTB to induce apoptosis of glioma cells. Collectively, the present study indicated that KHDRBS3 could regulate BTB permeability through the cDENND4C/miR-577 axis, which enhanced doxorubicin delivery across BTB. These findings may provide a novel strategy for chemotherapy of brain tumors.

## Introduction

Glioma is the most-common malignant central nervous system tumor^1^. Currently, the main treatment of glioma is surgery combined with postoperative chemoradiotherapy. Owing to the existence of blood–tumor barrier (BTB), macromolecular anti-tumor drugs are prevented from reaching brain tumor tissues. Therefore, the selective opening of BTB to increase the drug concentration in brain tumor tissues is very crucial to improve the effectiveness of chemotherapy for malignant glioma.

RNA-binding proteins (RBPs) form ribonucleoproteins complexes by binding to the target RNAs, and regulate gene expressions at the post-transcriptional levels, such as RNAs splicing, transport, stability, degradation, and translation^2–4^. RBPs are combined with non-coding RNAs to regulate their expressions and functions as well as involved in the occurrence and development of diseases^5^. RNA-binding protein KHDRBS3 (KH RNA-binding domain containing, signal transduction associated 3), belongs to the STAR (signal transduction and the activator of RNA) members of the family and regulates the selective splicing of target genes by binding to RNAs through the KH RNA-binding domain^6^. It is a tissue-specific RNA-binding protein, which is enriched in the brain. KHDRBS3 is also significantly upregulated in medulloblastoma cell lines, primary medulloblastoma tissues, breast cancer, and prostate cancer as well^7–9^. Quaking (QKI), another member of the STAR family, is not only involved in the formation and reconstruction of embryonic blood vessels, but also modulates the expression of VE-cadherin and β-catenin at the post-transcriptional level to affect the endothelial barrier function^10–12^.

Circular RNAs (circRNAs) are generated from reverse-cleaved or intron-derived covalently closed non-coding RNAs, without 5′ caps and 3′ poly (A) tails, and are tolerant to RNase R. CircRNA has a wide range of functions, for example, it acts as molecular sponges of miRNAs, regulates the expressions of parental genes, participates in the regulation of transcription and splicing^13^. CircRNAs are also involved in the regulation of vascular function^14,15^. In HUVECs, high glucose and hypoxic stress conditions increased the expression of circRNA ZNF609. Silencing of circRNA ZNF609 increased endothelial cells (ECs) migration and tubule formation and protected endothelial cells from oxidative and hypoxic stress in vivo^16^. Circular RNA DENND4C (cDENND4C, also known as hsa_circ_0005684), is derived from DENND4C gene exon 3–10. As a HIF1α-associated circRNA in breast cancer, the expression of cDENND4C is positively correlated with the breast tumor size^17^. Another study identified the endothelial circRNAs from a deep-sequencing experiment under the normoxic or hypoxic condition and confirmed that the expression of cDENND4C was also obviously increased after 12 h and 24 h of hypoxia^18^. We speculated that cDENND4C might be involved in the regulation of tumor vascular ECs function.

MicroRNAs (miRNAs) are a group of non-coding RNAs of about 22 nucleotides, which usually bind to the 3′ untranslated region (UTR) of mRNA, and negatively regulate expression of target genes at the post-transcriptional level. MiRNAs play important roles in biological function and are also involved in the regulation of BTB permeability^19,20^. Previous literature showed that miR-577 was downregulated in various types of tumors, including glioblastoma, hepatocellular carcinoma, colorectal cancer, et al.^21–24^. CircInteractome database (https://circinteractome.nia.nih.gov/) predicts that cDENND4C contains three binding sites of miR-577, suggesting that cDENND4C may act as a miR-577 sponge to bind to miR-577 and regulate the expression of its target gene. At present, the expression and function of miR-577 in GECs are not clear.

In this study, endogenous expressions of KHDRBS3, cDENND4C, and miR-577 in GECs were detected, and the interactions among them were analyzed to clarify the regulatory mechanisms for BTB permeability. This study confirmed that RNA-binding protein KHDRBS3 in GECs could bind to cDENND4C and increase its stability. cDENND4C affected the permeability of BTB by binding to and inhibiting the negative regulation of miR-577 on its target genes, which can provide a new strategy for the treatment of glioma.

## Result

### KHDRBS3 was highly expressed in GECs, and silencing of KHDRBS3 increased BTB permeability

**Fig. 1:**
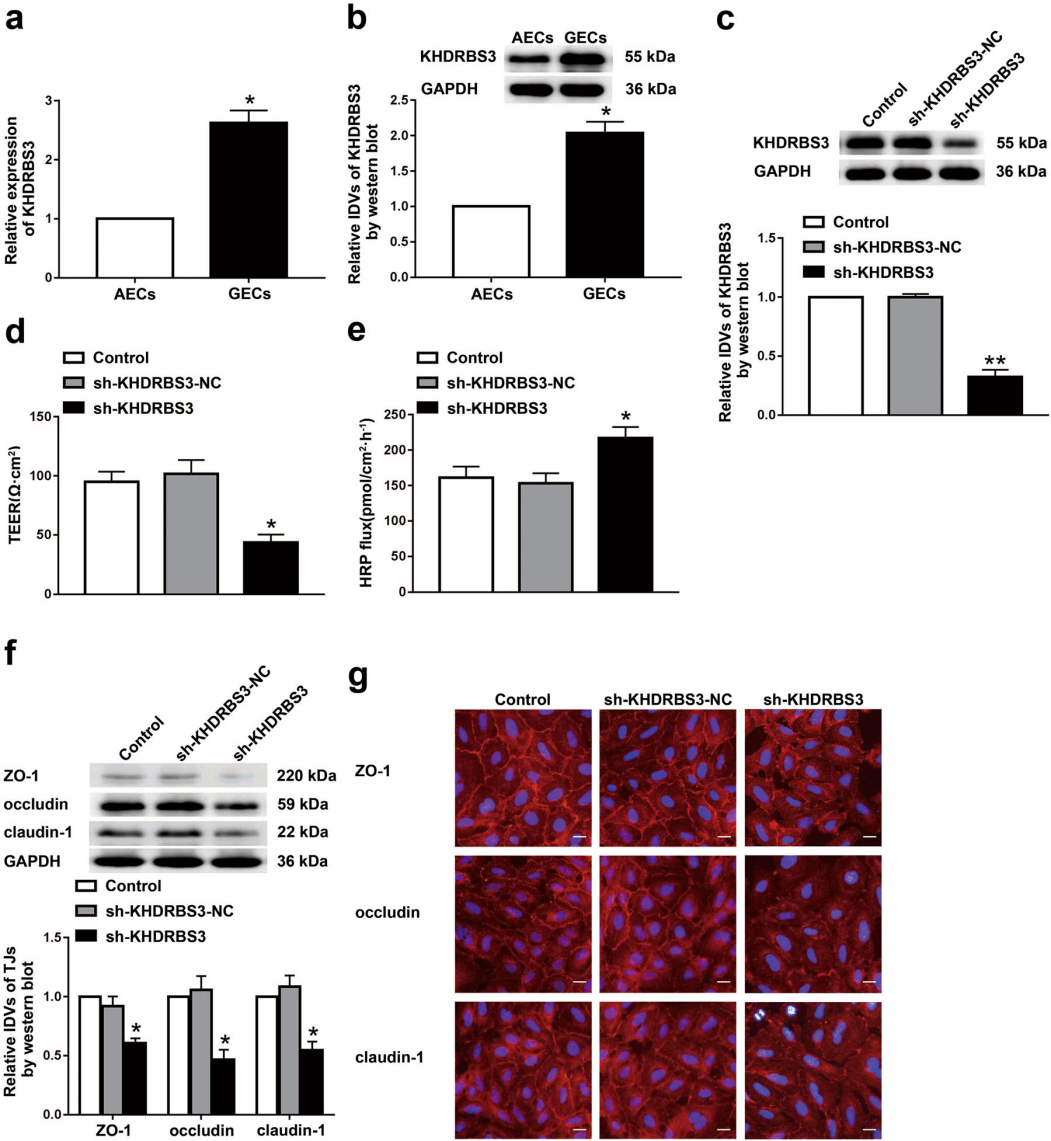
Knockdown of KHDRBS3 increased BTB permeability in vitro. **a** Relative mRNA levels of KHDRBS3 in AECs (the NHAs co-cultured ECs) and GECs (the glioma cells co-cultured ECs) were determined by qRT-PCR. **b** Relative protein levels of KHDRBS3 in AECs and GECs were determined by western blot. Data represented as mean ± SD (*n* = 3). *P < 0.05 vs. AEC group. **c** Relative expressive of KHDRBS3 was evaluated using western blot in the KHDRBS3 knockdown GECs. Data represented as mean ± SD (*n* = 3). **P < 0.01 vs. sh-KHDRBS3-NC group. The permeability and integrity of the KHDRBS3 knockdown BTB model in vitro were detected by TEER values **d** HRP flux **e**. **f** The expressions of ZO-1, occludin, and claudin-1 in the KHDRBS3 knockdown GECs detected by western blot. Data represented as mean ± SD (*n* = 3). *P < 0.05 vs. sh-KHDRBS3-NC group. **g** The distributions of ZO-1, occludin, and claudin-1 in the KHDRBS3 knockdown GECs were observed by immunofluorescent staining, (*n* = 3). Scale bar represents 50 µm

The RNA-binding protein KHDRBS3 belongs to the KH domain containing STAR family. The mRNA expression of KHDRBS3 showed the highest level among some of the family members in GECs from BTB models in vitro (Supplementary Fig. S1). As shown in Fig. 1a, b, the mRNA and protein expressions of KHDRBS3 in GECs were markedly increased compared with those in AECs from BBB models in vitro. After the knockdown, the KHDRBS3 expression in the sh-KHDRBS3 group decreased by 68% compared with the sh-KHDRBS3-NC group (Fig. 1c). When the BTB model in vitro was successfully constructed using stable KHDRBS3 knockdown ECs, TEER values, and horseradish peroxidase (HRP) flux were detected to analyze the integrity and permeability of BTB, respectively. As shown in Fig. 1d, e, compared with the sh-KHDRBS3-NC group, the TEER value in the sh-KHDRBS3 group was significantly decreased, and the HRP flux was markedly increased, indicating that KHDRBS3 was involved in the regulation of BTB permeability.

To clarify the underlying mechanism of KHDRBS3 in this process, the expression changes of tight junction-related protein ZO-1, occludin, and claudin-1 in KHDRBS3 knockdown GECs were detected (Fig. 1f). The results showed that compared with the sh-KHDRBS3-NC group, the expressions of ZO-1, occludin, and claudin-1 in the sh-KHDRBS3 group were significantly reduced. Moreover, consistent with the western blot results, the immunofluorescent staining indicated that the ZO-1, occludin, and claudin-1 in the control group and the sh-KHDRBS3-NC group showed relatively linear distributions on the tight junctions between GECs, whereas the continuous distributions of the above proteins was interrupted in the sh-KHDRBS3 group and the tight junctions between GECs were destroyed (Fig. 1g).

### cDENND4C was highly expressed in GECs, and cDENND4C knockdown increased BTB permeability

**Fig. 2:**
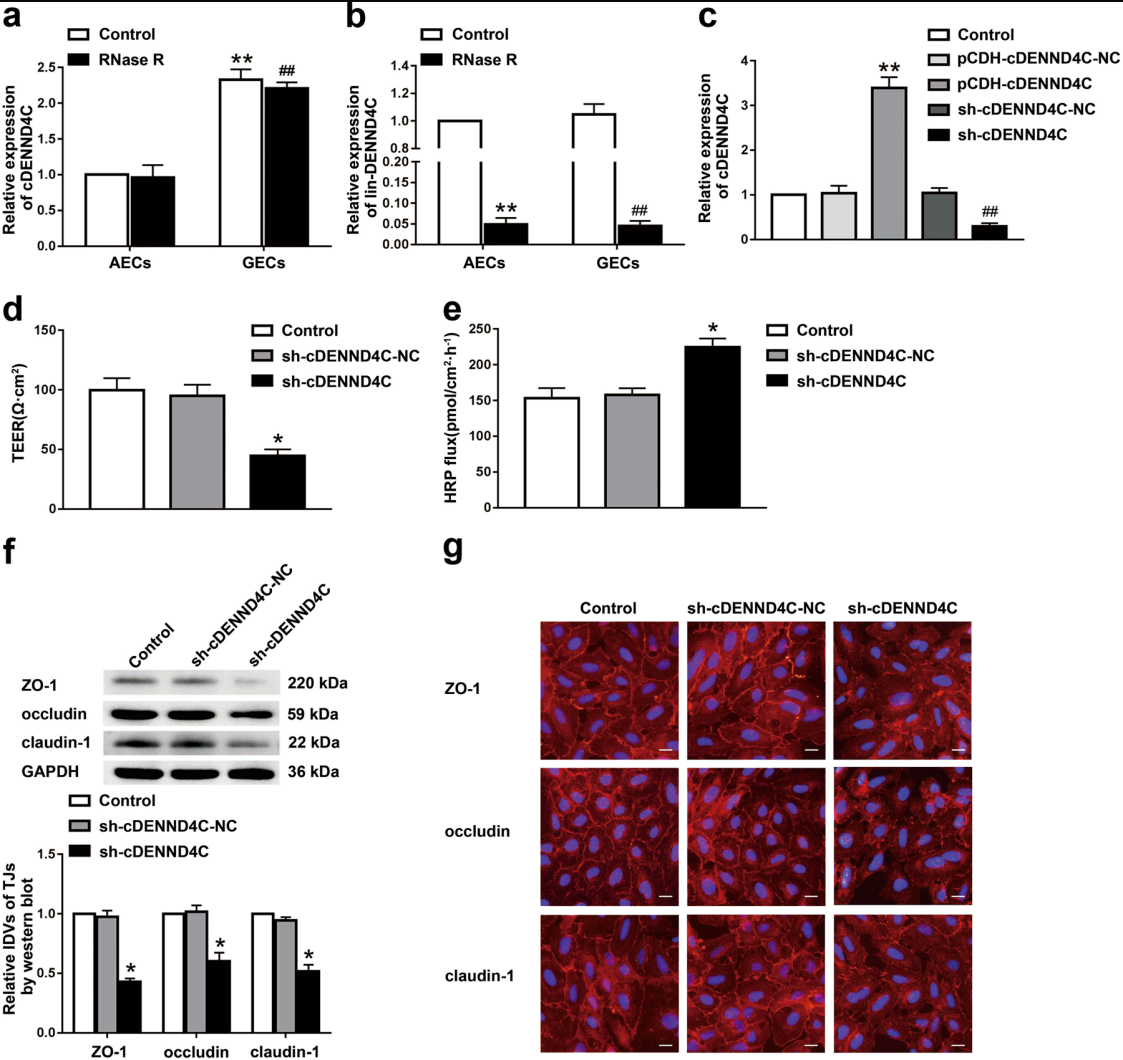
Knockdown of cDENND4C increased BTB permeability in vitro. **a** Relative expression of cDENND4C in AECs and GECs with RNase R treatment. Data represented as mean ± SD (*n* = 3). **P < 0.01 vs. AECs control group, ^##^*P* < 0.01 vs. AECs group with RNase R treatment. **b** Relative expression of lin-DENND4C in AECs and GECs with RNase R treatment. Data represented as mean ± SD (*n* = 3). **P < 0.01 or ^##^*P* < 0.01 vs. control group. **c** Relative expression of cDENND4C was evaluated using qRT-PCR in the cDENND4C overexpressed and knockdown GECs. Data represented as mean ± SD (*n* = 3). **P < 0.01 vs. pCDH-cDENND4C-NC group, ^##^*P* < 0.01 vs. sh-cDENND4C-NC group. The permeability and integrity of the cDENND4C knockdown BTB model were detected by TEER values **d** and HRP flux **e**. **f** The expressions of ZO-1, occludin, and claudin-1 in the cDENND4C knockdown GECs were detected by western blot. Data represented as mean ± SD (*n* = 3) *P < 0.05 vs. sh-cDENND4C-NC group. **g** The distributions of ZO-1, occludin, and claudin-1 in the cDENND4C knockdown GECs were observed by immunofluorescent staining, (*n* = 3). Scale bar represents 50 µm

The expression of cDENND4C in GECs was significantly upregulated compared with that in AECs (Fig. 2a). The circular properties of cDENND4C were verified using RNase R treatment. Compared with the control group, the expressions of cDENND4C in AECs and GECs remained unchanged after RNase R treatment. Compared with the AECs group, the expression of cDENND4C in the GECs group was markedly upregulated. Furthermore, no significant difference was observed in the expressions of lin-DENND4C between AECs and GECs groups. After RNase R treatment, the expressions of lin-DENND4C in AECs and GECs were markedly decreased compared with those in the control groups.

In order to investigate the effects of cDENND4C on BTB permeability, the BTB models in vitro were constructed using stably cDENND4C knocked-down or overexpressed ECs (Fig. 2c). Compared with the sh-cDENND4C-NC group, the TEER value of sh-cDENND4C group was significantly reduced, whereas the HRP flux was significantly increased (Fig. 2d, e), indicating that silence of cDENND4C destroyed the integrity of BTB and increased its permeability.

To further explore the potential mechanisms of cDENND4C in the regulation of BTB permeability, the expression changes of tight junction-related protein ZO-1, occludin, and claudin-1 in GECs after cDENND4C knockdown were detected. Compared with the sh-cDENND4C-NC group, the expressions of ZO-1, occludin, and claudin-1 in the sh-cDENND4C group were significantly reduced (Fig. 2f). In addition, the immunofluorescent staining also showed that the relatively continuous distributions of ZO-1, occludin, and claudin-1 in GECs was destroyed in the sh-cDENND4C group (Fig. 2g).

### KHDRBS3 affected the BTB permeability by regulating the expression of cDENND4C

**Fig. 3:**
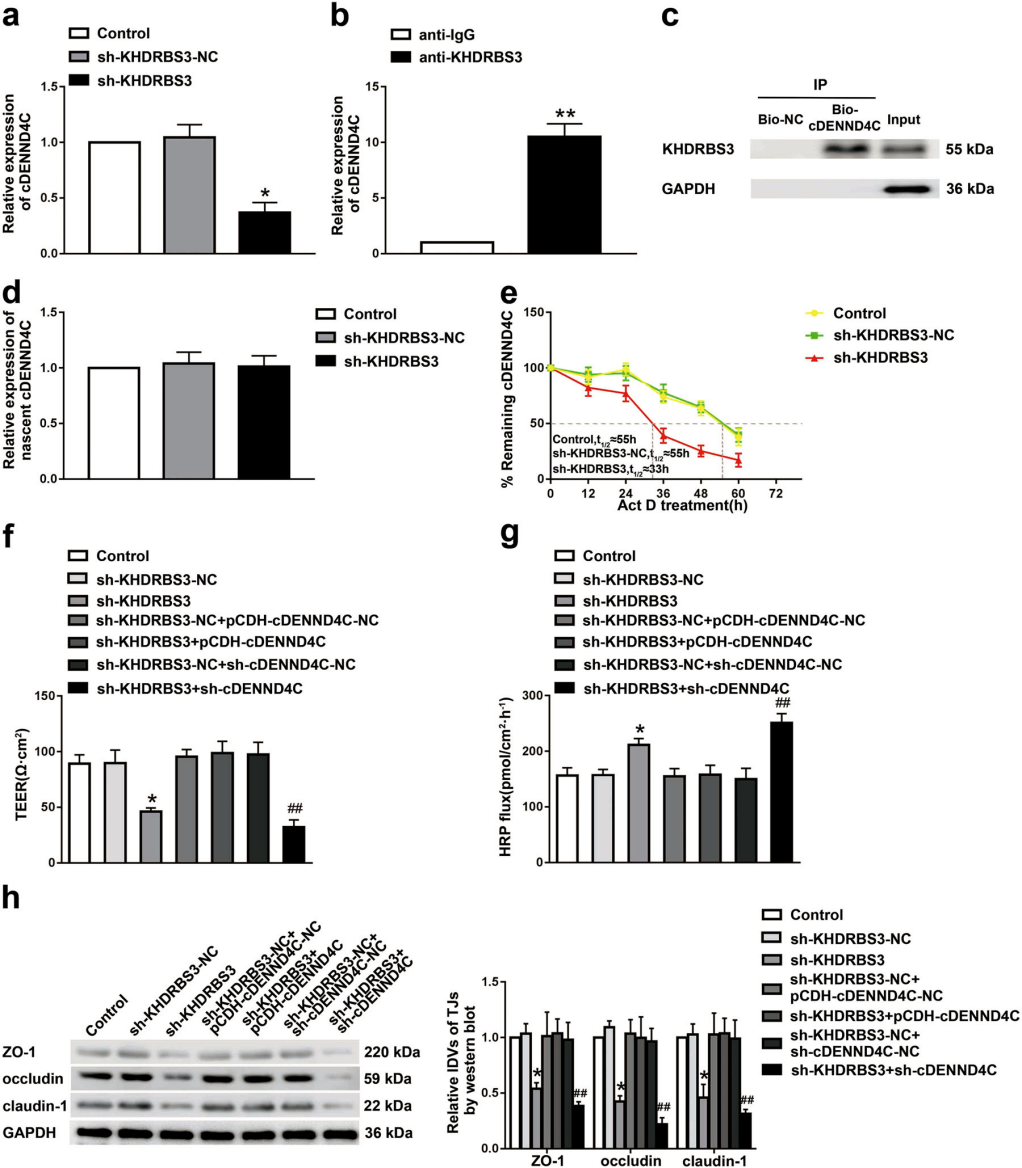
KHDRBS3 affected BTB permeability by regulating the expression of cDENND4C. **a** Relative expressive of cDENND4C was evaluated by qTR-PCR in the KHDRBS3 knockdown GECs. Data represented as mean ± SD (*n* = 3). *P < 0.05 vs. sh-KHDRBS3-NC group. **b** RNA immunoprecipitation assay was performed with normal mouse IgG or anti-KHDRBS3 antibody in GECs. Relative enrichment of cDENND4C was determined by qRT-PCR. Data represented as mean ± SD (*n* = 3). **P < 0.01 vs. anti-IgG group. **c** The retrieved KHDRBS3 protein levels with bio-cDENND4C and bio-NC were evaluated by western blot. **d** Relative expression levels of nascent cDENND4C in the KHDRBS3 knockdown GECs were detected using qRT-PCR. **e** Relative expression levels of cDENND4C in the KHDRBS3 knockdown GECs treated with actinomycin D at different time points were analyzed using qRT-PCR. The permeability and integrity of KHDRBS3 knockdown combined with cDENND4C overexpressed/knockdown BTB model in vitro were detected by TEER values **f** and HRP flux **g**. **h** The expressions of ZO-1, occludin, claudin-1 in the KHDRBS3 knockdown combined with cDENND4C overexpressed/knockdown GECs were detected by western blot. Data represented as mean ± SD (*n* = 3). *P < 0.05 vs. sh-KHDRBS3-NC group, ^##^*P* < 0.01 vs. sh-cDENND4C group

Using circBase (http://www.circbase.org) and RBP map database (http://rbpmap.technion.ac.il), we predicted the putative KHDRBS3-binding sites on the cDENND4C sequence. In order to uncover the molecular mechanisms of BTB permeability regulated by KHDRBS3 and cDENND4C, the expression level of cDENND4C in KHDRBS3 knockdown GECs was detected. As shown in Fig. 3a, compared with the sh-KHDRBS3-NC group, the expression of cDENND4C in the sh-KHDRBS3 group was downregulated. RIP indicated that KHDRBS3 could bind to cDENND4C. Compared with the IgG immunoprecipitates, the cDENND4C was significantly enriched in the KHDRBS3 immunoprecipitates (Fig. 3b). RNA pull-down results confirmed that the retrieved KHDRBS3 expressing level in the cDENND4C captured fraction was much higher than that in the bio-NC group, indicating that there was an interaction between cDENND4C and KHDRBS3 (Fig. 3c). After treated with RNase R, the nascent cDENND4C in the sh-KHDRBS3 group showed no significant difference with the sh-KHDRBS3-NC group. Moreover, the half-life of cDENND4C in the sh-KHDRBS3 group was shortened from 55 h to 33 h compared with the sh-KHDRBS3-NC group (Fig. 3d, e), suggesting that KHDRBS3 was involved in maintaining the stability of cDENND4C, but not nascence.

To clarify whether KHDRBS3 affect BTB permeability by regulating the expression of cDENND4C. First, stably KHDRBS3 knockdown ECs, KHDRBS3 knockdown and cDENND4C overexpressed ECs, KHDRBS3, and cDENND4C double knockdown ECs were established, respectively. After the in vitro BTB models were constructed, the expression levels of cDENND4C and KHDRBS3 were confirmed before the detection of the TEER and HRP flux of BTB (Supplementary Fig. S2). Compared with the sh-KHDRBS3-NC group, the TEER value in the sh-KHDRBS3 group was significantly decreased, and the HRP flux was significantly increased. Overexpression of cDENND4C could markedly reverse the reduction of barrier permeability induced by KHDRBS3 knockdown. However, double silence of KHDRBS3 and cDENND4C further downregulated the reduced barrier permeability induced by KHDRBS3 knockdown (Fig. 3f, g). In addition, cDENND4C overexpression significantly reversed the downregulated expressions of ZO-1, occludin, and claudin-1 caused by KHDRBS3 knockdown. The double knockdown of KHDRBS3 and cDENND4C further downregulated the reduced expressions of ZO-1, occludin, and claudin-1 caused by KHDRBS3 knockdown (Fig. 3h).

### MiR-577 was low expressed in GECs and was involved in the regulation of BTB permeability

**Fig. 4:**
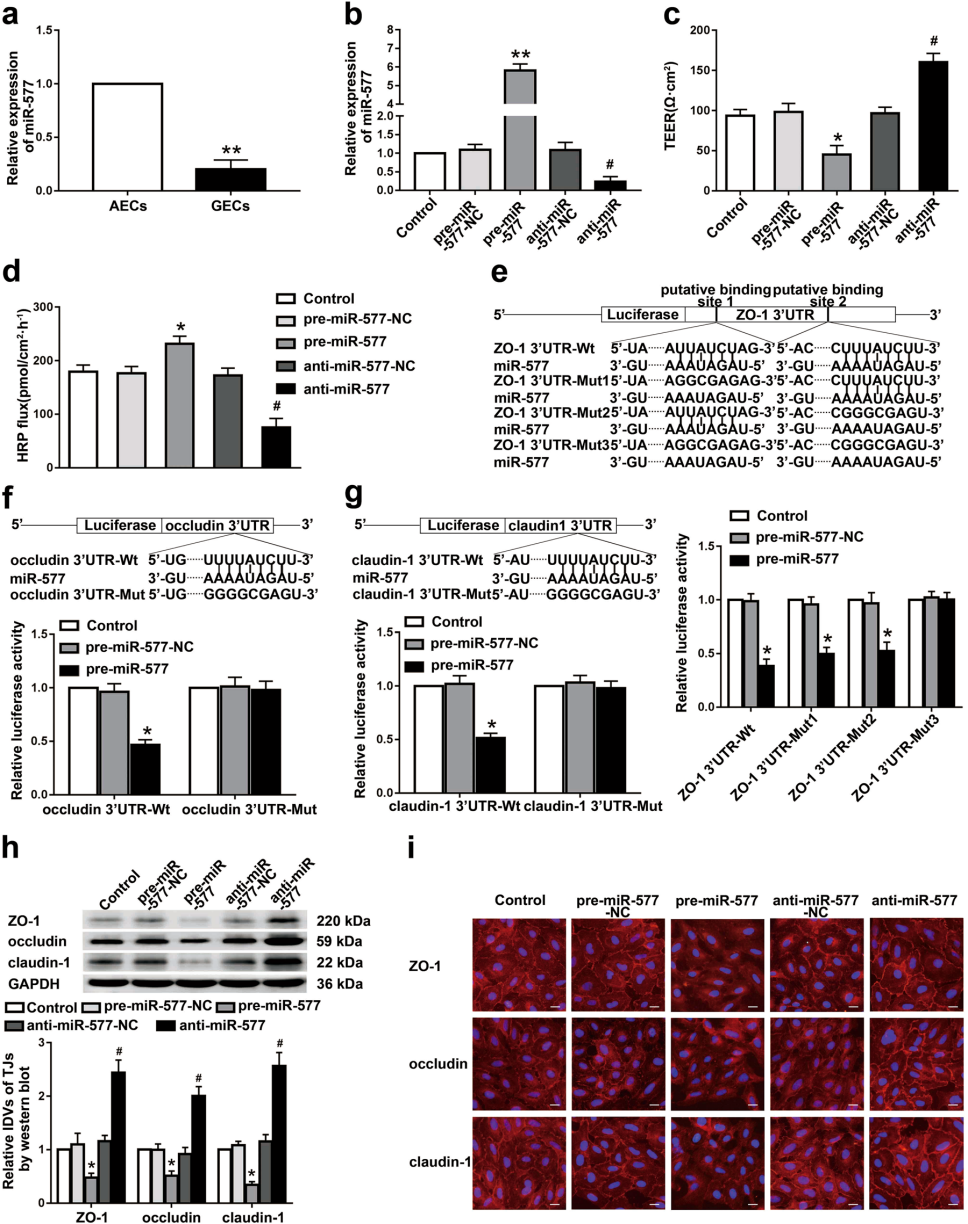
MiR-577 regulated the BTB permeability in vitro and the expressions of tight junction-related proteins in GECs. **a** Relative expression levels of miR-577 in AECs and GECs were determined by qRT-PCR. Data represented as mean ± SD (*n* = 3). **P < 0.01 vs. AECs group. **b** Relative expressions of miR-577 were evaluated using qRT-PCR in the miR-577 overexpressed or knockdown GECs. Data represented as mean ± SD (*n* = 3). **P < 0.01 vs. pre-miR-577-NC group, ^#^*P* < 0.05 vs. anti-miR-577-NC group. Effects of miR-577 expression changes on TEER values **c** and HRP flux **d** in vitro BTB model. Data represented as mean ± SD (*n* = 3). *P < 0.05 vs. pre-miR-577-NC group, ^#^*P* < 0.05 vs. anti-miR-577-NC group. The putative miR-577 binding sites in the 3′-UTR of ZO-1 mRNA **e**, occludin mRNA **f**, claudin-1 mRNA **g** and designed mutant sequences are presented. Relative luciferase activity was performed by dual-luciferase reporter assays. Data represented as mean ± SD (*n* = 3). *P < 0.05 vs. pre-miR-577-NC group. **h** Effects of miR-577 expression changes on the ZO-1, occludin and claudin-1 protein levels were detected by Western blot. Data represented as mean ± SD (*n* = 3). *P < 0.05 vs. pre-miR-577-NC group, ^#^*P* < 0.05 vs. anti-miR-577-NC group. **i** Effects of miR-577 expression changes on the distributions of ZO-1, occludin, and claudin-1 were detected by immunofluorescent staining, (*n* = 3). Scale bar represents 40 µm

Compared with the AECs group, the expression of miR-577 in the GECs group was significantly decreased (Fig. 4a). After knockdown and overexpression of miR-577 in ECs (Fig. 4b), the effects of miR-577 expression on the permeability and integrity of BTB were evaluated. Compared with the pre-miR-577-NC group, the TEER value of the pre-miR-577 group was significantly decreased, and the HRP flux was significantly increased. Compared with the anti-miR-577-NC group, the TEER value of the anti-miR-577 group was significantly increased, and the HRP flux was significantly decreased (Fig. 4c, d), suggesting that miR-577 was involved in the regulation of BTB permeability.

Using bioinformatics database TargetScan Human 7.2 (http://www.targetscan.org/vert_72/), we predicted two miR-577 binding sites at the 3′-UTR of ZO-1 mRNA, one miR-577-binding site at the 3′-UTR of occludin mRNA, and one miR-577-binding site at the 3′-UTR of claudin-1 mRNA. The dual-luciferase reporter assay confirmed that compared with the control group, the luciferase activities of pre-miR-577 and ZO-1 3′-UTR-Wt/-Mut1/-Mut2 double transfection group were significantly reduced. However, compared with the control group, the luciferase activity of pre-miR-577 and ZO-1 3′-UTR-Mut3 double-transfection group showed no significant change (Fig. 4e). Compared with the control group, the pre-miR-577 and occludin 3′-UTR-Wt double transfection group showed lower luciferase activity. However, there was no significant difference in the luciferase activity between the pre-miR-577 and occludin 3′-UTR-Mut double transfection group and the control group (Fig. 4f). Compared with the control group, the luciferase activity of pre-miR-577 and claudin-1 3′-UTR-Wt double transfection group was significantly reduced. However, there was no significant change in the luciferase activity between the pre-miR-577 and claudin-1 3′-UTR-Mut double transfection group and the control group (Fig. 4g). The above results indicate that miR-577 could bind to the 3′-UTRs of ZO-1, occludin, and claudin-1 mRNA, respectively.

In order to explore the mechanism of miR-577 involved in regulating BTB permeability, the effects of miR-577 knockdown or overexpression on tight junction-related protein ZO-1, occludin, and claudin-1 in GECs were detected. The results showed that compared with the pre-miR-577-NC group, the expressions of ZO-1, occludin, and claudin-1 in the pre-miR-577 group were significantly reduced. Compared with the anti-miR-577-NC group, the expressions of the above proteins in the anti-miR-577 group were markedly increased (Fig. 4h). Immunofluorescent staining results showed that compared with the pre-miR-577-NC group, the relatively continuous distributions of ZO-1, occludin, and claudin-1 of the pre-miR-577 group in GECs disappeared, whereas the continuous distributions of ZO-1, occludin, and claudin-1 of the anti-miR-577 group in GECs became more linear (Fig. 4i).

### cDENND4C interacted with miR-577 to regulate the permeability of BTB

**Fig. 5:**
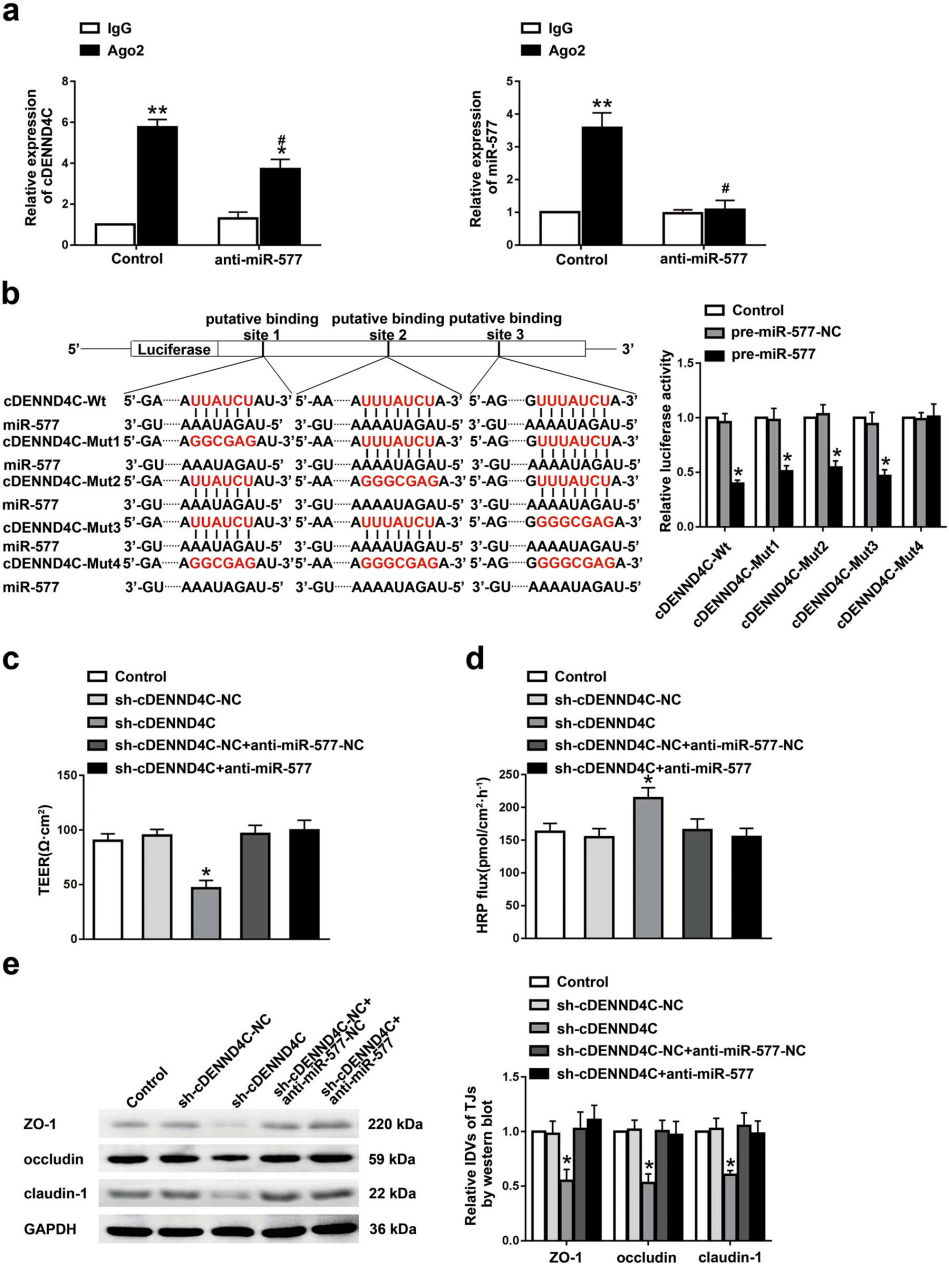
cDENND4C interacted with miR-577 to regulate BTB permeability. **a** RNA immunoprecipitation assay was performed with normal mouse IgG or Ago2 antibody in miR-577 knockdown GECs. Relative enrichment of cDENND4C and miR-577 was determined by qRT-PCR. Data represented as mean ± SD (*n* = 3). **P < 0.01 vs. IgG group, *P < 0.05 vs. IgG group, ^#^*P* < 0.05 vs. Ago2 group. **b** The putative miR-577-binding sites in the cDENND4C and designed mutant sequences are presented. Relative luciferase activity was performed by dual-luciferase reporter assays. Data represented as mean ± SD (*n* = 3). *P < 0.05 vs. pre-miR-577-NC group. Effects of cDENND4C and miR-577 double knockdown on TEER values **c** and HRP flux **d** in vitro BTB model. **e** Effects of cDENND4C and miR-577 double knockdown on the expressions of ZO-1, occludin, claudin-1 were analyzed by western blot. Data represented as mean ± SD (*n* = 3). *P < 0.05 vs. sh-cDENND4C-NC group

Using CircInteractome database (https://circinteractome.nia.nih.gov/), we predicted three miR-577-binding sites on the cDENND4C sequence and presumed that cDENND4C might function as a molecular sponge of miR-577 to regulate the BTB permeability. To verify this hypothesis, the interaction between cDENND4C and miR-577 was detected. RIP results showed that cDENND4C was significantly enriched in Ago2 group compared with IgG group. After knockdown of miR-577, the enrichment level of cDENND4C in Ago2 group decreased significantly (Fig. 5a). The result of the dual-luciferase reporter assay was consistent with our expectation. Compared with the control group, the pre-miR-577 and cDENND4C-Wt/-Mut1/-Mut2/-Mut3 double transfection group showed lower luciferase activity. However, there was no significant change in the luciferase activity between the pre-miR-577 and cDENND4C-Mut4 double transfection group and the control group (Fig. 5b). To verify whether the interaction between cDENND4C and miR-577 could affect the permeability of BTB, the TEER value and HRP flux in vitro BTB models with the cDENND4C knockdown, and cDENND4C and miR-577 double knockdown were detected, respectively. The results showed that cDENND4C and miR-577 double knockdown could significantly reverse the decreased TEER value and increased HRP flux induced by cDENND4C knockdown (Fig. 5c, d). Figure 5e showed that compared with the cDENND4C knockdown group, the expressions of ZO-1, occludin, and claudin-1 were significantly increased in the cDENND4C and miR-577 double knockdown group, indicating that cDENND4C and miR-577 double knockdown reversed the downregulated tight junction-related proteins induced by cDENND4C knockdown, which was consistent with the change tendency of barrier permeability.

### Combined treatment of KHDRBS3, cDENND4C, and miR-577 promoted doxorubicin (DOX) delivery across BTB to induce apoptosis of glioma cells

**Fig. 6:**
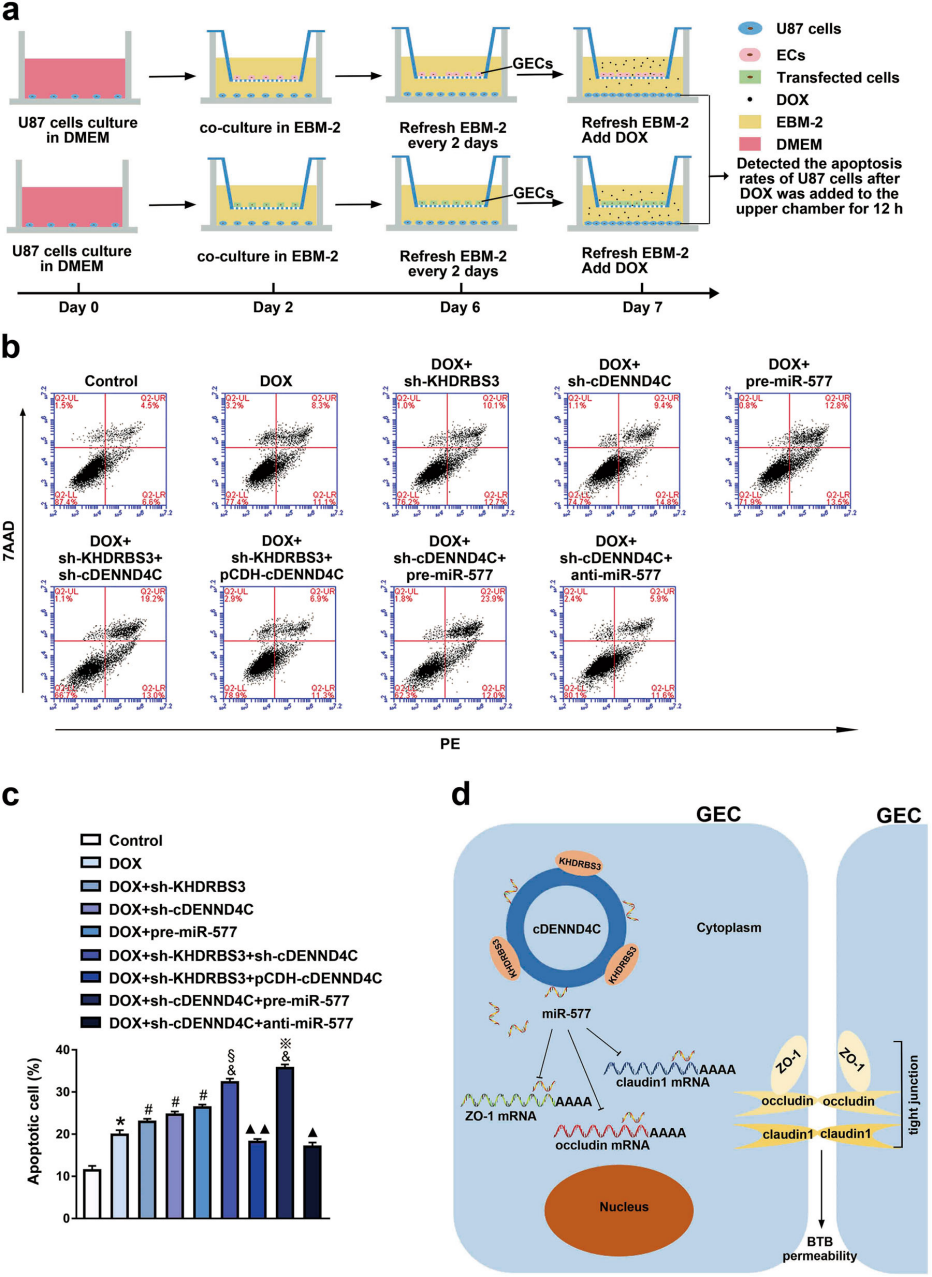
The apoptosis changes of U87 cells induced by combined treatment of KHDRBS3, cDENND4C, and miR-577 with doxorubicin. **a** The schematic diagram of the BTB model in vitro used to evaluate the Dox penetration. Representative flow cytometric detection **b** and analysis of the U87 cell apoptosis rates in different groups **c**. Data represented as mean ± SD (*n* = 3). *P < 0.05 vs. control group, ^#^*P* < 0.05 vs. DOX group, ^§^*P* *<* 0.05 vs. DOX + KHDRBS3 knockdown GECs group, ^&^*P* *<* 0.05 vs. DOX + cDENND4C-knockdown GECs group, ^※^*P* *<* 0.05 vs. DOX + miR-577 overexpressed GECs group, ^▲▲^*P* *<* 0.01 vs. DOX + KHDRBS3 and cDENND4C double knockdown GECs group, ^▲^*P* < 0.01 vs. DOX + cDENND4C knockdown combined with miR-577 overexpressed GECs group. **d** The schematic diagram of the mechanism by which KHDRBS3 regulates the BTB permeability via cDENND4C/miR-577 axis

The anti-tumor drug DOX has been shown to poorly cross the BBB and not to penetrate the brain tumor cells because of multidrug resistance mechanisms. In order to verify whether KHDRBS3, cDENND4C, and miR-577 in GECs might become the potential targets to increase the BTB permeability and improve the DOX across BTB to better induce the apoptosis of U87 glioma cells, the apoptosis rates of U87 cells in the following groups were detected, respectively. Before the apoptosis analysis, the expression levels of KHDRBS3, cDENND4C and miR-577 were confirmed after the BTB models were established (Supplementary Fig. S3). A schematic diagram of the BTB model in vitro used to evaluate the Dox penetration was shown in Fig. 6a. Compared with the DOX group, the apoptosis rate of U87 cells was significantly higher in the KHDRBS3- or cDENND4C-knockdown GECs or miR-577 overexpressed GECs treated with DOX group. Moreover, compared with KHDRBS3 or cDENND4C single knockdown GECs treated with DOX group, the apoptosis rate of U87 cells was increased markedly in the double knockdown of KHDRBS3 and cDENND4C genes in GECs treated with DOX group. Furthermore, the overexpression of cDENND4C in the KHDRBS3 knockdown GECs treated with DOX significantly reversed the upregulated U87 cell apoptosis rate in the KHDRBS3 and cDENND4C double knockdown GECs treated with DOX. Compared to cDENND4C knockdown or miR-577 overexpressed GECs treated with DOX group, the apoptosis rate of U87 cells was significantly increased in the cDENND4C knockdown combined with miR-577 overexpressed GECs treated with DOX group. Whereas, the overexpression of miR-577 in the cDENND4C-knockdown GECs treated with DOX significantly reversed the upregulated U87 cell apoptosis rate in the cDENND4C and miR-577 double knockdown GECs treated with DOX. The above results indicate that the GECs with KHDRBS3 and cDENND4C double knockdown, and the GECs with miR-577 overexpression combined with cDENND4C knockdown showed better DOX penetration across BTB than the GECs with single transfection of those three factors (Fig. 6b, c). A schematic representation of the mechanism by which the KHDRBS3/cDENND4C/miR-577 axis regulates the BTB permeability is presented in Fig. 6d.

## Discussion

Many linear non-coding RNAs and circRNAs can be used as scaffolding to bind with multiple RBPs, such as AGO, thus increasing the stability of circRNAs transcripts^25,26^. KHDRBS3 belongs to RBPs of STAR family containing the KH domain. Members of this family are mainly involved in the regulation of selective splicing, export, and stability of RNAs^6^. Studies have shown that QKI, another member of this family, is the main regulator of de novo circRNAs formation during human epithelial–mesenchymal transition^27^. In our research, we found that the expression level of KHDRBS3 in GEC was obviously higher than part of the family members, such as QKI. After silencing of KHDRBS3 in GEC, the integrity of BTB was damaged and the permeability was increased, suggesting that KHDRBS3 might be involved in the regulation of GEC functions. At present, there are few studies focused on the regulatory functions of RBPs about vascular ECs. Previous studies confirmed that HuR bound to Sirtuin 1 and stabilized its mRNA expression, which inhibited the release of E-selectin and activation of endothelial cells induced by TNF-α and high sugar^28^. QKI maintained endothelial barrier integrity by binding to the mRNAs of VE-cadherin and β-catenin and promoting their translations^11^. Furthermore, RIP and RNA pull-down experiments proved that KHDRBS3 could bind to cDENND4C. cDENND4C was enriched in GECs. Silencing of cDENND4C in GECs destroyed the integrity of BTB by reducing the expression of tight junction-related proteins. After knockout of KHDRBS3 in ECs, the expression level of cDENND4C was significantly reduced and its half-life was shortened as well, suggesting that KHDRBS3 may regulate the permeability of BTB by binding to cDENND4C and increase its stability. Other studies confirmed that RNA-binding proteins MOV10 and FUS also bound to circRNAs and positively regulated their expressions to promote the angiogenesis of gliomas, which is consistent with our results. All the above data suggest that RBPs could affect the functions of vascular ECs by regulating the expression of certain non-coding RNAs^29,30^.

In recent years, accumulating evidence has shown that circRNAs play an important regulatory role in ECs functions, and the mechanism is mainly related to the role of miRNA molecular sponges in inhibiting the negative regulation of miRNAs on their target genes. The overexpressed circDLGAP4 in ECs acted as the sponge of miR-143 to inhibit its activity and upregulated the expression its target gene HECTD1, which inhibited the endothelial–mesenchymal transformation, alleviated the defects of cerebral neurons, and reduced infarct area and blood-brain barrier damage^31^. In GECs, circ_002136 bound to miR-138–5p and inhibited its negative regulation of transcription factor SOX13, formed the FUS/circ_002136/miR-138–5p/SOX13 feedback loop to regulate the proliferation, migration, and tube-formation of GECs^30^. In addition, the abnormal expressions of circRNAs also affect the occurrence and development of gliomas. CircNT5E could be used as a molecular sponge of miR-422a to regulate the proliferation, apoptosis, migration, and invasion of glioma cells^32^. CircPINTexon2 encoded an 87-aa tumor suppressive peptide, which played a glioma-suppressive role by interacting with PAF1 protein^33^. Under the hypoxic condition, the expression of cDENND4C in ECs was significantly increased, suggesting that cDENND4C is a HIF1 α-associated circRNAs^17,18^. In this study, the cDENND4C was highly expressed in GECs, which acted as the sponge of miR-577 to inhibit the negative regulation of its target gene and further affected the permeability of BTB. This suggests that cDENND4C is a kind of circRNAs closely related to the regulation of ECs functions under hypoxia or tumor condition. The expression levels of lin-DENND4C showed no difference in AECs and GECs, suggesting the expression levels of cDENND4C and lin-DENND4C were not correlated, which was inconsistent with the results of He Q and Zheng J et al.^34,35^.

More and more studies have confirmed that the dysregulated miRNAs in vascular ECs not only affected the proliferation and migration of endothelial cells but were also involved in the regulation of endothelial barrier permeability. For example, miR-27a-3p was downregulated in the serum of patients with cerebral hemorrhage, which upregulated aquaporin 11 to increase the permeability of cerebral microvascular ECs, destroyed the blood-brain barrier and caused cerebral edema^36^. MiR-577, as a tumor suppressor gene, is related to the development of a variety of cancers. In colorectal cancer, the lowly expressed miR-577 bound the 3′-UTR of target gene HSP27 to negatively regulate its expression and affected the proliferation and migration of colorectal cancer cells^22^. In the present study, miR-577 was lowly expressed in GECs. Overexpression or silencing of miR-577 in GECs increased or decreased the permeability of BTB, suggesting that miR-577 contributed to the regulation of endothelial barrier function. Numerous studies have confirmed that the distributions of tight junction-related protein ZO-1, occludin and claudin-1 in brain microvascular ECs are closely related to the permeability of the blood-brain barrier^37,38^. Double luciferase reporter gene assays demonstrated that miR-577 bound the 3′-UTR of ZO-1, occludin and claudin-1 mRNAs to negatively regulate the expression of the above proteins, suggesting that miR-577 can directly regulate the expression of ZO-1, occludin, and claudin-1, and affect the permeability of BTB.

Dox, a class of anthracyclines anti-tumor antibiotics, is widely used in the clinical treatment of various types of cancers^39^. For the treatment of brain tumors, owing to the existence of the blood-brain barrier, it is difficult for Dox to enter the brain and reach an effective therapeutic concentration. To further evaluate the regulatory effect of BTB permeability by treatment of KHDRBS3, cDENND4C, and miR-577 alone or in combination, Dox was combined with the above regulatory factors. It was demonstrated that double silencing of KHDRBS3 and cDENND4C or silencing of cDENND4C and overexpression of miR-577 significantly increased the apoptosis rate of U87 glioma cells induced by Dox, compared with the silencing of KHDRBS3, cDENND4C, or overexpression of miR-577 alone, suggesting that the treatment of KHDRBS3, cDENND4C, and miR-577 alone or in combination could enhance the antitumor effect of Dox by promoting its penetrating capability across BTB.

In summary, the upregulated KHDRBS3 in GECs affected the permeability of BTB by binding to cDENND4C and increasing its stability. cDENND4C, acted as a miR-577 sponge, bound to and inhibited the negative regulation of miR-577 on the tight junction-related protein ZO-1, occludin and claudin-1 to affect the BTB permeability. Single or combined treatment of KHDRBS3, cDENND4C, and miR-577 effectively promote the anti-tumor effect of Dox. These studies can provide new therapeutic strategies for glioma.

## Materials and methods

### Cell lines and cell culture

The immortal human brain EC line hCMEC/D3 was provided by Dr. Couraud (Cochin Institute, Paris, France). ECs were cultured on Transwell permeable support systems (0.4-μm pore size; Corning, Lowell, MA, USA) coated with Cultrex Rat Collagen I at 150 μg/mL (R&D Systems, Minneapolis, MN, USA). The culture medium contained endothelial basal medium (Lonza, Walkersville, MD, USA) containing 5% fetal bovine serum “Gold” (PAA Laboratories, Pasching, Austria), in which 1% penicillin–streptomycin (Life Technologies, Paisley, UK), 1% chemically defined lipid concentrate (Life Technologies, Paisley, UK), 1.4 µm hydrocortisone (Sigma-Aldrich, St Louis, MO, USA), 1 ng/mL human basic fibroblast growth factor (Sigma-Aldrich), 5 µm ascorbic acid (Sigma-Aldrich), and 10 mm HEPES (PAA Laboratories). ECs were limited from 30 to 40 passages. Human brain astrocytoma cell line U87MG (U87) and human embryonic kidney cell line HEK-293T (293T) were purchased from the Shanghai Institutes for Biological Sciences Cell Resource Center. The cells were cultured with Dulbecco's Modified Eagle Medium (DMEM) culture medium containing 10% calf serum, 100 U/ml penicillin and 100 µg/ml streptomycin (Life Technologies, Paisley, UK). Normal human astrocytes cell line (NHAs) were purchased from Sciencell Research Laboratories (Carlsbad, CA, USA) and were cultured in astrocyte medium RPMI-1640 (GIBCO, Carlsbad, CA, USA). All cells were maintained at 37 °C in a humidified incubator of 5% CO_2_.

### Establishment of In Vitro BTB and BBB model

In vitro BTB and BBB models were established by co-culture of ECs, U87 cells and NHA cells as described previously^25^. For the BTB model, the U87 cells were seeded onto the 6-well culture plate at a density of 2 × 10^4^ per well for 2 days. Then, the ECs were seeded onto the Transwell insert pretreated with 150 µg/ml Cultrex Rat Collagen I (R&D Systems, Minneapolis, MN, USA) at a density of 2 × 10^5^ per well. The inserts were placed in the well of the six-well plates containing U87 glioma cells and co-cultured with EBM-2 medium for 4 days, and the medium was changed every 2 days. The glioma cells co-cultured ECs were called GECs. For the BBB model, the NHAs were seeded onto the six-well culture plate at a density of 2 × 10^4^ per well for 2 days. And the subsequent process is the same as the BTB model in vitro. The NHAs co-cultured ECs were called AECs.

### qRT-PCR assay

Total RNAs were extracted by Trizol reagent (Life Technologies, Carlsbad, CA, USA), and the purity and concentration of RNAs were determined by a Nanodrop Spectrophotometer (ND-100, Thermo Scientific, Waltham, MA). One-Step SYBR PrimeScript RT-PCR Kit (Perfect Real Time) (RR066A, TakaraBio, Japan) was used to detect the expression of QKI5, QKI6, QKI7, KHDRBS1, KHDRBS2, KHDRBS3, cDENND4C, and linear DENND4C. RNase R was used to confirm the cDENND4C, eliminated the influence of linear RNAs. GAPDH was used as the endogenous control. TaqMan MicroRNA Reverse Transcription kit and TaqMan Universal Master Mix II (Applied Biosystems, Foster City, CA, USA) was used to detect the expression of miR-577. U6 was used as the endogenous control. Relative expression values were normalized and calculated using the relative quantification (2^-△△Ct^) method. Primers used in this study are shown in Supplementary Table S1.

### Cell transfection

Short-hairpin RNA (shRNA) direct against human KHDRBS3 gene was ligated into pGPU6/RFP/Hygro vector (JTS Scientific, Wuhan, China) to silence KHDRBS3. Silencing plasmid of cDENND4C (sh-cDENND4C) was constructed using pGPU6/GFP/Neo vector (GenePharma, Shanghai, China). The full-length cDENND4C sequence was cloned into a pCDH-CMV-MCS-EF1-Puro vector (Geneseed Biotech, Guangzhou, China) to obtain the cDENND4C overexpression plasmid (pCDH-cDENND4C). The pGPU6/RFP/Hygro, pGPU6/GFP/Neo, and pCDH-CMV-MCS-EF1-Puro empty vectors were used as the negative control (NCs). The ECs were seeded in the 24-well plate with 80% confluence then transfected with the sh-KHDRBS3 plasmid, sh-cDENND4C plasmid and their respective NCs using Opti-MEM^®^ I and Lipofectamine LTX Reagent (Life Technologies, Carlsbad, CA, USA). The stably KHDRBS3 or cDENND4C knockdown ECs were selected by using the culture medium containing 0.4 mg/mL Hygromycin (Solarbio, Beijing, China) and 0.4 mg/mL of Geneticin (G418, Sigma-Aldrich, St Louis, MO, USA), respectively for ~ 4 weeks. To obtain the stably cDENND4C overexpressed ECs, the lentivirus packaging plasmid (Invitrogen, Waltham, MA, USA) and pCDH-cDENND4C expression plasmids were co-transfected into HEK-293T cells, using Lipofectamine 3000 (Invitrogen, Thermo Fisher Scientific, USA). The lentiviruses with a minimum titer of 5 × 10^5^ infectious units/mL were harvest and used to infect ECs for 2 days. 0.5 μg/ml of puromycin was used for selection of the stably cDENND4C overexpressed ECs. The KHDRBS3 and cDENND4C double knockdown ECs as well as the KHDRBS3 knockdown with cDENND4C overexpressed ECs were established using KHDRBS3 knockdown plasmid to transfect into cDENND4C silenced or overexpressed ECs and were selected with culture media containing Hygromycin and G418 or puromycin. The stably transfected cells with Hygromycin and G418 or puromycin resistance were obtained after 4 weeks. Cell transfection efficiencies were determined by qRT-PCR and western blot. The sequences of sh-KHDRBS3, sh-cDENND4C, and sh-NC were shown in Supplementary Table S2.

AgomiR-577, antagomiR-577, and their respective NC were transfected transiently into the ECs using the Lipofectamine 3000 reagent. For the co-transfection of sh-cDENND4C and antagomiR-577, the antagomiR-577 were transfected transiently into the stably cDENND4C knockdown ECs. All cells were obtained 48 h after transient transfection. Cells transfection efficiencies were determined by qRT-PCR.

### Transendothelial electric resistance (TEER) assay

The TEER values were detected after the establishment of the BTB models in vitro using a Millicell-ERS instrument (Millipore, Billerica, MA, USA). In order to remove the influences of temperature and medium on the measurement results, equal amounts of the new medium should be replaced in the upper and lower chambers of Transwell and TEER value was measured at room temperature for 30 min. The final resistance (Ω cm^2^) was calculated by subtracting background resistance from measured barrier resistance and then multiplied by the effective surface area of the filter membrane.

### HRP flux assay

After the establishment of BTB models in vitro, 1 ml of serum-free EBM-2 medium containing 10 µg/ml HRP (0.5 μm, Sigma-Aldrich, USA) was added into the upper chamber of Transwell, and 2 ml of complete medium was added into the lower chamber. After incubated at 37 °C for 1 h, 5 µl of culture medium from the lower chamber was collected and analyzed using tetramethylbenzidine colorimetry approach with a spectrophotometer at 370 nm. The final HRP flux was calculated from the standard curve and expressed as pmol passed per cm^2^ surface area per hour (pmol/cm^2^ h^−1^).

### Western blot assay

Total proteins of cells were extracted using RIPA buffer (Beyotime Institute of Biotechnology, Jiangsu, China) supplemented with protease inhibitors (10 mg/mL aprotinin, 10 mg/mL PMSF, and 50 mm sodium orthovanadate). Then, the BCA protein assay kit (Beyotime Institute of Biotechnology, Jiangsu, China) was used to determine the protein concentration. The same amount of proteins (40 µg) were loaded for SDS–PAGE electrophoresis, and then transferred to polyvinylidene difluoride membrane (Millipore, Shanghai, China) and sealed in Tris-buffered saline/Tween 20 (TBST) containing 5% skimmed milk powder at room temperature for 2 h. The primary antibodies to incubate the membrane are as follows: KHDRBS3 (1:1 000; Proteintech, Chicago, IL, USA), GAPDH (1:10 000; Proteintech, Chicago, IL, USA), ZO-1 (1:300; Life Technologies, Frederick, MD, USA), occludin (1:1 000; Proteintech, Chicago, IL, USA), and claudin-1 (1:300; Life Technologies, Frederick, MD, USA) at 4 °C overnight. The membranes were washed three times with TBST and then incubated with HRP-conjugated secondary antibody (1:5000; Santa Cruz Biotechnology, Santa Cruz, CA, USA) at room temperature for 2 h. Protein blots were revealed by an enhanced chemiluminescence kit (ECL; Santa Cruz Biotechnology, Dallas, TX) and detected by ECL Detection Systems (Thermo Scientific, Beijing, China), then scanned using Chemi Imager 5500 V2.03. Integrated density value (IDV) of the bands was quantitatively analyzed by Fluor Chen2.0, and GAPDH was used as an internal reference to determine the expression level of the target protein.

### Immunofluorescence assay

The cells were fixed with 4% paraformaldehyde at room temperature for 30 min and permeated in PBS containing 0.2% Triton X-100 for 10 min (ZO-1 and claudin-1) or fixed with methanol for 10 min at − 20 °C (occludin). After washed with PBS for three times, cells were sealed with 5% BSA at room temperature for 2 h. The primary antibodies used to incubate the cells were as follows: ZO-1 (1:50; Life Technologies), occludin (1:50; Abcam), and claudin-1 (1:50; Life Technologies) at 4℃ overnight. The next day, after washing with PBS/Tween 20 (PBST) for three times, the cells were incubated with Alexa-Fluor-555-labeled goat anti-rabbit or anti-mouse immunofluorescence secondary antibody (1:500; Beyotime Institute of Biotechnology, Jiangsu, China) at room temperature in the dark for 2 h. Then the nuclei were stained with 0.5 µg/mL of DAPI for 5 min. The staining was observed using immunofluorescence microscopy (Olympus, Tokyo, Japan).

### Reporter vector construction and dual-luciferase reporter assay

The potential binding sequence and the corresponding mutant sequence of miR-577 in cDENND4C, ZO-1 3′-UTR, occludin 3′-UTR and claudin-1 3′-UTR were amplified by PCR and cloned into the pmirGLO Dual-Luciferase miRNA Target Expression Vector (Promega, Madison, WI, USA) to construct wild type and mutation type luciferase reporter vectors (Generay Biotech Co., Shanghai, China). HEK-293T cells were co-transfected with the above wild type and mutation type luciferase reporter vectors and the pre-miR-577 or pre-miR-577-NC using Lipofectamine 3000. Then, the luciferase activities were analyzed 48 h after transfection using the Dual-Luciferase reporter kit (Promega, Madison, WI, USA), and the relative luciferase activity was expressed as the ratio of firefly luciferase activity to renilla luciferase activity.

### RNA immunoprecipitation (RIP) assay

Whole cells lysates of different groups were collected and incubated overnight with magnetic bead RIP buffer containing anti-human argonaute 2 (Ago2) antibody (Millipore, Billerica, MA, USA) and anti-human KHDRBS3 antibody (Proteintech). The negative control group was incubated with normal mouse IgG (Millipore). Samples were incubated with Proteinase K buffer and then immunoprecipitated RNA was isolated. The RNA concentration was determined by a spectrophotometer (NanoDrop, Thermo Scientific), and the RNA quality was assessed using a biological analyzer (Agilent, Santa Clara, CA, USA). Then RNA was purified, and reverse transcribed. The RNA enrichment was assessed by qRT-PCR.

### RNA pull-down assay

The interaction between KHDRBS3 and cDENND4C was tested by Pierce Magnetic RNA-Protein Pull-Down Kit (Thermo Fisher) according to the manufacturer’s instruction. In brief, Biotin-labeled cDENND4C (bio-cDENND4C) were in vitro transcribed with the Biotin RNA Labeling Mix (Roche) and T7 RNA polymerase (Promega), treated with RNase-free DNase I (Promega) and purified with RNeasy Mini Kit (QIAGEN). The bio-cDENND4C and bio-antisense RNA (NC) were incubated with GECs lysates followed by extensive washes. Magnetic beads were added to prepare a probe–magnetic bead complex. The retrieved proteins were analyzed by Western blot with GAPDH as the control.

### Nascent RNA capture

According to the manufacturer’s protocol, nascent RNA capture assay was performed using the Click-iT^®^ Nascent RNA Capture Kit (Thermo Fisher Scientific, USA). In brief, KHDRBS3 knockdown cells and its negative control were incubated with 5-ethynyl uridine (EU) and total RNA labeled with EU was isolated using TRIzol reagent (Invitrogen) and treated with RNase R. EU-nascent circular RNA was biotinylated in a Click-iT reaction buffer and captured using streptavidin magnetic beads for qRT-PCR analysis.

### RNA stability measurement

Cells were cultured in the medium containing 5 µg/ml actinomycin D (Act D, NobleRyder, China) to block the de novo RNA synthesis. Total RNA was extracted from the cells collected at 12, 24, 36, 48, 60 h. The expression of cDENND4C was detected by qRT-PCR. The half-life of cDENND4C was detected by its expressing level at a certain time point compared with time zero.

### Analysis of apoptosis by flow cytometry

The BTB models were established in vitro using the ECs with KHDRBS3 knockdown, cDENND4C knockdown, miR-577 overexpression, KHDRBS3, and cDENND4C double knockdown, KHDRBS3 knockdown combined with cDENND4C overexpression, cDENND4C knockdown combined with miR-577 overexpression, cDENND4C and miR-577 double knockdown. After the BTB model in vitro was established, 10 µm of DOX (Beyotime Institute of Biotechnology, Jiangsu, China) was added to the upper chamber of Transwell. The apoptosis rates of U87 cells seeded in the lower chamber were detected 12 h later by using the Annexin V-PE/7AAD kit (Southern Biotech, Birmingham, AL, USA). The U87 cells in the lower chamber were resuspended with Annexin V bounding buffer after washed with phosphate-buffered saline and centrifuging twice. Resuspended cells were stained with Annexin V-PE/7AAD for 15 min in the dark at room temperature. Cell samples were obtained by FACScan (BD Biosciences) to obtain the apoptotic fractions and analyzed by CELL Quest 3.0 software. Early apoptosis is defined by Annexin V^+^/PI^−^ staining (lower right quadrant, LR), and late apoptosis is defined by Annexin V^+^/PI^+^ staining (upper right quadrant, UR).

### Statistical analysis

All the experiments in this study were repeated at least three times independently. Data were presented in the form of the mean ± SD. GraphPad Prism v5.01 software was used for analysis. Student’s *t* test was used to determine the difference between the two groups. One-way analysis of variance followed by Bonferroni’s post hoc test was used to determine the difference among multiple groups. *P* < 0.05 was considered to be statistically significant.

## Supplementary information

Supplementary Data.

## References

[1] Zhu D (2017). Up-regulation of miR-497 confers resistance to temozolomide in human glioma cells by targeting mTOR/Bcl-2. Cancer Med..

[2] Castello A (2012). Insights into RNA biology from an atlas of mammalian mRNA-binding proteins. Cell.

[3] Dreyfuss G, Kim VN, Kataoka N (2002). Messenger-RNA-binding proteins and the messages they carry. Nat. Rev. Mol. Cell Biol..

[4] Wang ZL (2018). Comprehensive genomic characterization of RNA-binding proteins across human cancers. Cell Rep..

[5] Darnell RB (2010). RNA regulation in neurologic disease and cancer. Cancer Res. Treat..

[6] Vernet C, Artzt K (1997). STAR, a gene family involved in signal transduction and activation of RNA. Trends Genet..

[7] Lu Y (2009). Amplification and overexpression of Hsa-miR-30b, Hsa-miR-30d and KHDRBS3 at 8q24.22-q24.23 in medulloblastoma. PLoS ONE.

[8] Matsumoto Y, Itou J, Sato F, Toi M (2018). SALL4 - KHDRBS3 network enhances stemness by modulating CD44 splicing in basal-like breast cancer. Cancer Med..

[9] Lei KF (2011). SerpinB5 interacts with KHDRBS3 and FBXO32 in gastric cancer cells. Oncol. Rep..

[10] Chenard CA, Richard S (2008). New implications for the QUAKING RNA binding protein in human disease. J. Neurosci. Res..

[11] de Bruin RG (2016). The RNA-binding protein quaking maintains endothelial barrier function and affects VE-cadherin and β-catenin protein expression. Sci. Rep..

[12] Noveroske JK (2002). Quaking is essential for blood vessel development. Genesis.

[13] Li X, Yang L, Chen LL (2018). The biogenesis, functions, and challenges of circular RNAs. Mol. Cell.

[14] Cheng X, Joe B (2017). Circular RNAs in rat models of cardiovascular and renal diseases. Physiol. Genomics.

[15] Li CY, Ma L, Yu B (2017). Circular RNA hsa_circ_0003575 regulates oxLDL induced vascular endothelial cells proliferation and angiogenesis. Biomed. Pharmacother..

[16] Liu C (2017). Silencing of circular RNA-ZNF609 ameliorates vascular endothelial dysfunction. Theranostics.

[17] Liang G (2017). HIF1α-associated circDENND4C promotes proliferation of breast cancer cells in hypoxic environment. Anticancer Res..

[18] Boeckel JN (2015). Identification and characterization of hpoxia-regulated endothelial circular RNA. Circ. Res..

[19] Guo J (2017). Long non-coding RNA NEAT1 regulates permeability of the blood-tumor barrier via miR-181d-5p-mediated expression changes in ZO-1, occludin, and claudin-5. Biochim. Biophys. Acta Mol. Basis Dis..

[20] Liu J (2017). The role of miR-330-3p/PKC-α signaling pathway in low-dose endothelial-monocyte activating polypeptide-II increasing the permeability of blood-tumor barrier. Front. Cell Neurosci..

[21] Wang LY, Li B, Jiang HH, Zhuang LW, Liu Y (2015). Inhibition effect of miR-577 on hepatocellular carcinoma cell growth via targeting β-catenin. Asian Pac. J. Trop. Med..

[22] Wang Y (2018). Long noncoding RNA DANCR promotes colorectal cancer proliferation and metastasis via miR-577 sponging. Exp. Mol. Med..

[23] Xue KC, Hu DD, Zhao L, Li N, Shen HY (2017). MiR-577 inhibits papillary thyroid carcinoma cell proliferation, migration and invasion by targeting SphK2. Eur. Rev. Med. Pharmacol. Sci..

[24] Yin C (2018). MiR-577 suppresses epithelial-mesenchymal transition and metastasis of breast cancer by targeting Rab25. Thorac. Cancer.

[25] Cai H (2015). Roundabout 4 regulates blood-tumor barrier permeability through the modulation of ZO-1, Occludin, and Claudin-5 expression. J. Neuropathol. Exp. Neurol..

[26] Jeck WR, Sharpless NE (2014). Detecting and characterizing circular RNAs. Nat. Biotechnol..

[27] Conn SJ (2015). The RNA binding protein quaking regulates formation of circRNAs. Cell.

[28] Ceolotto G (2014). Sirtuin 1 stabilization by HuR represses TNF-alpha- and glucose-induced E-selectin release and endothelial cell adhesiveness in vitro: relevance to human metabolic syndrome. Clin. Sci. (Lond).

[29] He Q (2019). MOV10 binding circ-DICER1 regulates the angiogenesis of glioma via miR-103a-3p/miR-382-5p mediated ZIC4 expression change. J. Exp. Clin. Cancer Res..

[30] He Z (2019). FUS/circ_002136/miR-138-5p/SOX13 feedback loop regulates angiogenesis in Glioma. J. Exp. Clin. Cancer Res..

[31] Bai Y (2018). Circular RNA DLGAP4 ameliorates ischemic stroke outcomes by targeting miR-143 to regulate endothelial-mesenchymal transition associated with blood-brain barrier integrity. J. Neurosci..

[32] Wang R (2018). CircNT5E acts as a sponge of miR-422a to promote glioblastoma tumorigenesis. Cancer Res..

[33] Zhang M (2018). A peptide encoded by circular form of LINC-PINT suppresses oncogenic transcriptional elongation in glioblastoma. Nat. Commun..

[34] He Q (2018). Circ-SHKBP1 regulates the angiogenesis of U87 glioma-exposed endothelial eells through miR-544a/FOXP1 and miR-379/FOXP2 pathways. Mol. Ther. Nucleic Acids.

[35] Zheng J (2017). TTBK2 circular RNA promotes glioma malignancy by regulating miR-217/HNF1β/Derlin-1 pathway. J. Hematol. Oncol..

[36] Xi T (2018). MiR-27a-3p protects against blood-brain barrier disruption and brain injury after intracerebral hemorrhage by targeting endothelial aquaporin-11. J. Biol. Chem..

[37] Chen L (2018). MiR-429 regulated by endothelial monocyte activating polypeptide-II (EMAP-II) influences blood-tumor barrier permeability by inhibiting the expressions of ZO-1, Occludin and Claudin-5. Front. Mol. Neurosci..

[38] Sladojevic N (2019). Claudin-1-dependent destabilization of the blood-brain barrier in chronic stroke. J. Neurosci..

[39] Gabizon AA, Patil Y, La-Beck NM (2016). New insights and evolving role of pegylated liposomal doxorubicin in cancer therapy. Drug Resist. Updat..

