# Supplementary material for: KHDRBS3 regulates the permeability of blood–tumor barrier via cDENND4C/miR-577 axis
Source: Cell Death Dis. 2019 Jul 11;10(7):536. doi: 10.1038/s41419-019-1771-2 (PMC6624200; doi:10.1038/s41419-019-1771-2)
Supplement: Supplementary file 1 — Supplementary Data. [file 41419_2019_1771_MOESM1_ESM.pdf]

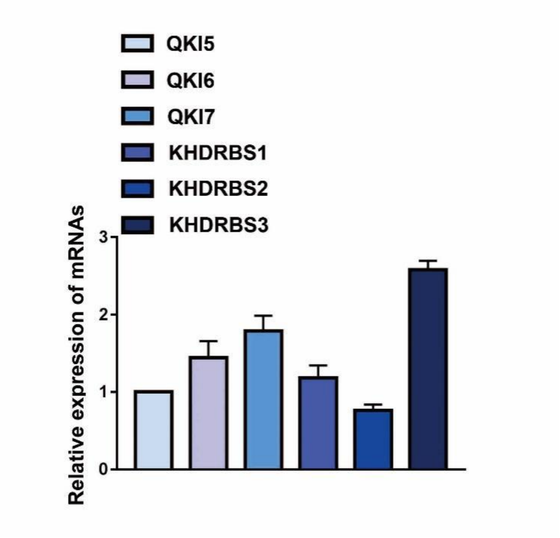

Fig. S1 Relative expressions of QKI5, QKI6, QKI7, KHDRBS1, KHDRBS2 and KHDRBS3 mRNAs were evaluated by qPCR in the GECs. Data represented as mean ± SD (n = 3).

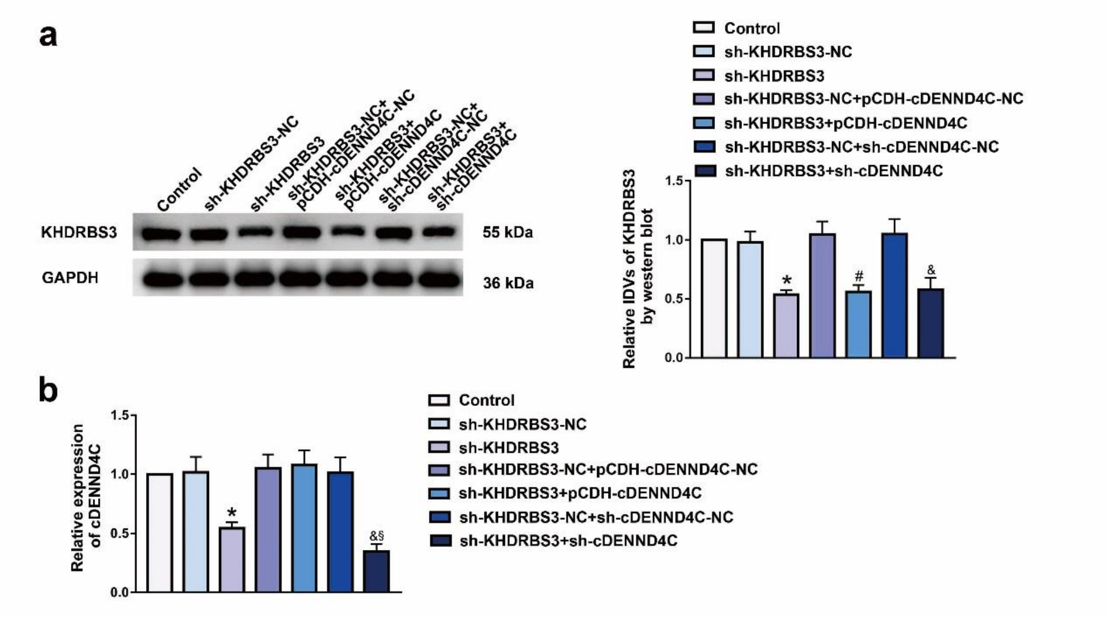

Fig. S2 The expression of KHDRBS3 was analyzed by Western blot **(a)** and the expression of cDENND4C was detected by qPCR **(b)** in the KHDRBS3 knockdown GECs, the KHDRBS3 knockdown combined with cDENND4C overexpressed GECs and the KHDRBS3 and cDENND4C double-knockdown GECs. Data represented as mean ± SD (n = 3). $^{*}P<0.05$ vs. sh-KHDRBS3-NC group, $^{\#}P<0.05$ vs. sh-KHDRBS3-NC+pCDH-cDENND4C-NC group, $^{\&}P<0.05$ vs. sh-KHDRBS3-NC+sh-cDENND4C-NC group, $^{\S}P<0.05$ vs. sh-KHDRBS3 group.

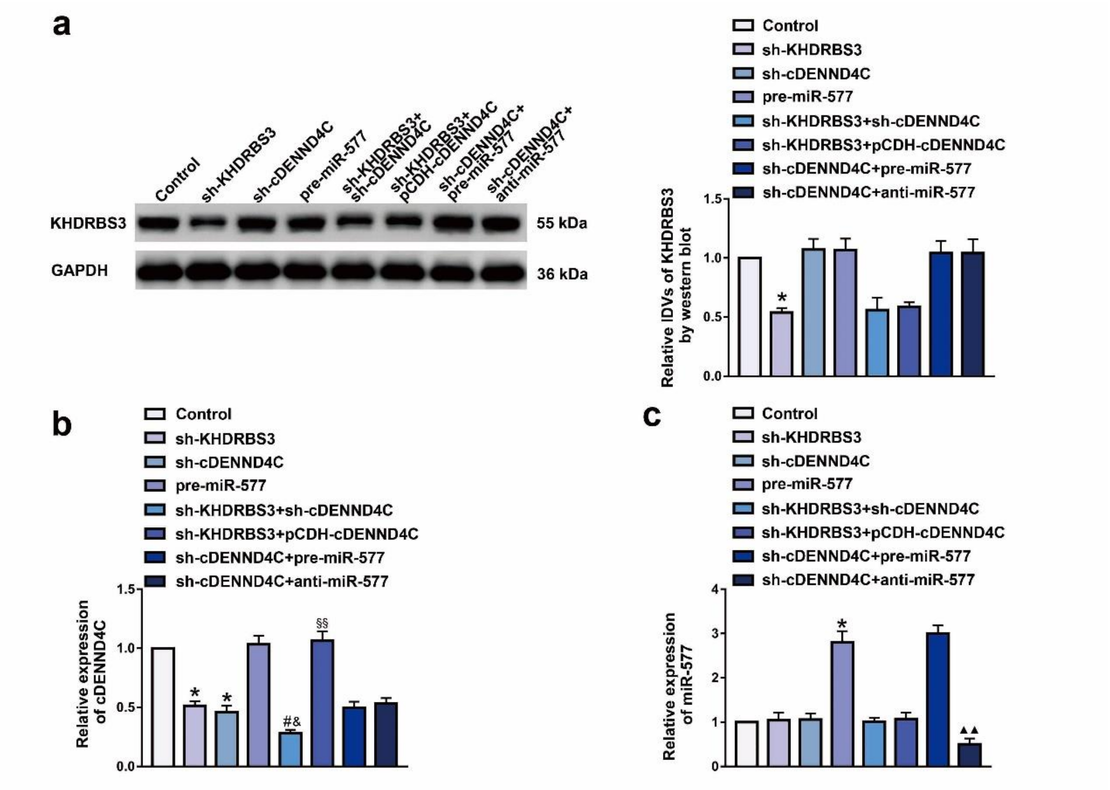

Fig. S3 The expressions of KHDRBS3, cDENND4C and miR-577 were analyzed by Western blot **(a)** and qPCR **(b, c)** in the GECs with KHDRBS3, cDENND4C or miR-577 knockdown/overexpressed alone or in combination. Data represented as mean ± SD (n = 3). **P*<0.05 vs. control group, #*P*<0.05 vs. sh-KHDRBS3 group, &*P*<0.05 vs. sh-cDENND4C group, §§*P*<0.01 vs. sh-KHDRBS3+sh-cDENND4C group, ▲▲*P*<0.01 vs. sh-cDENND4C+pre-miR-577 group.

Table. S1 Primers used for qRT-PCR

Gene	Sequence (5'->3') or Assay ID
cDENND4C	F: CTTCATGACCCACCACAAGATG
	R: GGGTGTGGCTAGGATCACTTC
lin-DENND4C	F: ACCCAGAGCAACACAAGTCT
	R: ACATCAAAACCTCCATTGCACA
KHDRBS1	F: GCCACAGCCTCGGTCAAGATG
	R: AGCTGCATGGCGTGAGTGAAG
KHDRBS2	F: TGGTCGTGGCAGAGGTATTAGAGG
	R: CTTGCTACAGGTGGCACTGGAAG
KHDRBS3	F: ACGGACATGGACTCAGTGAGGAG
	R: CTCGCTGAAGGTGCCTTGTGTC
QKI5	F: AATCCTTGAGTATCCTATTGAACCTAGT
	R: GCATATCGTGCCTTCGAACTTT
QKI6	F: AGCTACATCAATCCTTGAGTATCCTATTG
	R: TAGCCTTTCGTTGGGAAAGC
QKI7	F: GCTACATCAATCCTTGAGTATCCTATTG
	R: CAGGCATGACTGGCATTTCA
GAPDH	F: GGTGAAGGTCGGAGTCAACG
	R: CCATGTAGTTGAGGTCAATGAAG
MiR-577	002675 (Applied Biosystems)
U6	001973 (Applied biosystems)

Table. S2 Sequences of shRNA template

Gene		Sequence (5'->3')
KHDRBS3	Sence	CACCGGGACATGCTTTGGAAGAAATTCAAGAGATTTCTTCCAAAGCATGTCCTTTTTTG
	Antisence	GATCCAAAAAAGGACATGCTTTGGAAGAAATCTCTTGAATTTCTTCCAAAGCATGTCCC
cDENND4C	Sence	CACCGAACATGTTATATGTAGTTCTTTCAAGAGAAGAACTACATATAACATGTTCTTTTTTG
	Antisence	GATCCAAAAAAGAACATGTTATATGTAGTTCTTCTCTTGAAAGAACTACTATAACATGATC
NC	Sence	CACCGTTCTCCGAACGTGTCACGTCAAGAGATTACGTGACACGTTCGGAGAATTTTTTG
	Antisence	GATCCAAAAAAGTTCTCCGAACGTGTCACGTAATCTCTTGACGTGACACGTTCGGAGAAC
